# Genotype-Phenotype Correlations in Pediatric Hereditary Pancreatitis: Evidence from a Romanian Retrospective Cohort

**DOI:** 10.3390/jcm15051779

**Published:** 2026-02-26

**Authors:** Alexandra Coroleucă, Corina-Valentina Dragu, Roxana-Elena Matran, Irina Dijmărescu, Raluca Maria Vlad, Ioana Anca Bădărău, Cristina-Adriana Becheanu

**Affiliations:** 1 Department of Pediatrics, Faculty of Medicine, Carol Davila University of Medicine and Pharmacy, Dionisie Lupu Street, No. 37, 020021 Bucharest, Romania; alexandra.coroleuca@umfcd.ro (A.C.); rsmadeanu@yahoo.com (R.-E.M.); irina.dijmarescu@umfcd.ro (I.D.); raluca.vlad@umfcd.ro (R.M.V.); anca.badarau@umfcd.ro (I.A.B.); cbecheanu@yahoo.com (C.-A.B.); 2 Pediatrics, Emergency Hospital for Children Grigore Alexandrescu, 30-32 Iancu de Hunedoara Boulevard, 011743 Bucharest, Romania

**Keywords:** hereditary pancreatitis, pediatric pancreatitis, *PRSS1*, *SPINK1*, *CFTR*, genetic mutations, chronic pancreatitis

## Abstract

**Objectives**: This study aimed to evaluate the genetic and clinical characteristics of hereditary pancreatitis (HP) in a cohort of Romanian pediatric patients, with a focus on the correlation between specific gene mutations and disease severity, recurrence patterns, and associated complications. **Methods**: A retrospective analysis was conducted on pediatric patients diagnosed with HP. Genetic testing was performed to identify mutations in key genes. Clinical data were collected regarding age of onset, recurrence, severity, surgical interventions, and progression to chronic pancreatitis. Exploratory computational analyses were performed to illustrate potential patterns between genetic variants and clinical characteristics. **Results**: *PRSS1* variants were associated with earlier onset and more severe disease. A substantial proportion of patients developed chronic pancreatitis during the study period. Surgical complications were observed in over half of the cohort. The findings are consistent with the role of genetic mutations in influencing the clinical trajectory of HP. Genotype–phenotype correlations may inform hypotheses regarding early risk stratification and tailored management strategies. **Conclusions**: Genetic testing is essential for the accurate diagnosis and personalized treatment of HP. Integrating genetic diagnostics into clinical practice may improve outcomes and guide early intervention in high-risk pediatric patients.

## 1. Introduction

Hereditary pancreatitis (HP) represents a rare but clinically significant cause of acute pancreatitis (AP), acute recurrent pancreatitis (ARP), and chronic pancreatitis (CP) in pediatric patients [1]. Population-based data from high-income countries estimate an overall HP prevalence of approximately 0.3–0.57 per 100,000, confirming its status as a true rare disease [1]. HP is classically characterized by an autosomal dominant inheritance pattern with variable penetrance and is most frequently associated with pathogenic variants in genes involved in pancreatic enzyme regulation, particularly *PRSS1*. Additional pancreatitis-related genes, including *SPINK1*, *CFTR*, *CTRC*, and *CPA1*, are generally considered susceptibility or disease-modifying genes rather than classical causative genes, contributing to variable penetrance and phenotypic heterogeneity in pediatric pancreatitis [1,2].

In children, early recognition of HP remains challenging because clinical manifestations are often non-specific and overlap with more common environmental or acquired etiologies, such as biliary disease, trauma, infections, drug-induced pancreatitis, metabolic disorders, or structural anomalies. As a result, underdiagnosis and diagnostic delays are frequent, especially in the absence of a clearly documented family history. Nevertheless, establishing a positive family history and confirming the presence of pathogenic genetic mutations remain essential diagnostic criteria [3].

HP is notable for its early onset and its strong propensity for progression from AP or ARP to CP. This progression is associated with substantial long-term morbidity, including exocrine pancreatic insufficiency, diabetes mellitus, and an increased lifetime risk of pancreatic adenocarcinoma [1]. In pediatric CP cohorts, hereditary pancreatitis has been reported to account for approximately 10–20% of cases, underscoring its clinical relevance in this age group [4]. These outcomes highlight the importance of early diagnosis and comprehensive management strategies tailored to the underlying genetic profile.

In Romania, the landscape of genetic testing for HP and other rare diseases has evolved considerably in recent years. The increasing availability of molecular diagnostic techniques has improved the detection of causative mutations and enabled more personalized approaches to care. However, despite these advances, significant challenges persist. Limited resources and disparities in access—particularly in rural or under-resourced regions—continue to restrict timely genetic evaluation. Moreover, due to constraints in local infrastructure and the availability of specialized expertise, certain genetic analyses still require outsourcing to laboratories abroad, leading to potential delays in diagnosis and initiation of appropriate management strategies. These limitations emphasize the ongoing need to integrate comprehensive genetic testing into routine clinical practice and to ensure equitable access across different healthcare settings.

Given the scarcity of national data, the literature on genotype–phenotype correlations in pediatric HP is largely dominated by international cohorts, while Romanian pediatric experience remains limited. Therefore, the objective of this study was to investigate the genetic and clinical characteristics of hereditary pancreatitis in a Romanian pediatric population, with particular emphasis on the distribution of pancreatitis-associated gene variants and their relationship with age at onset, recurrence patterns, disease severity, complications, and progression to CP. Considering the exploratory nature and small cohort size, the analyses were designed to be hypothesis-generating rather than confirmatory, aiming to contribute to improved diagnostic precision, risk stratification, and individualized management of affected children.

## 2. Materials and Methods

This retrospective observational cohort study included pediatric patients hospitalized at “Grigore Alexandrescu” Children’s Emergency Clinical Hospital (Bucharest, Romania) between January 2022 and December 2025 and diagnosed with acute pancreatitis (AP), acute recurrent pancreatitis (ARP), or chronic pancreatitis (CP), as defined by the INSPPIRE criteria [5]. According to these criteria, AP was defined by the presence of at least two of the following: (i) abdominal pain consistent with pancreatitis, (ii) serum amylase and/or lipase levels ≥ 3 times the upper limit of normal, and (iii) imaging findings characteristic of pancreatitis. ARP was defined as two or more distinct episodes of AP with complete resolution of symptoms and normalization of pancreatic enzymes between episodes. CP was defined by irreversible structural changes of the pancreas, documented by imaging and/or histology, associated with chronic pancreatic inflammation and resulting in exocrine and/or endocrine dysfunction [5].

Eligible patients were aged <18 years at first presentation and had a positive pancreatitis-related genetic test identifying at least one pathogenic or likely pathogenic variant in a pancreatitis-associated gene. We acknowledge that while *PRSS1* variants are classically associated with autosomal dominant hereditary pancreatitis, variants in genes such as *SPINK1*, *CFTR*, *CTRC*, and *CPA1* are more commonly considered disease-modifying or susceptibility factors rather than independent causative mutations [1]. Cases were identified through systematic review of hospital discharge diagnoses and electronic medical records.

Exclusion criteria included pancreatitis attributable to acute abdominal trauma, drug-induced pancreatitis, hypertriglyceridemia or hypercalcemia, gallstone disease as the primary etiology, structural pancreaticobiliary anomalies explaining the episodes (e.g., pancreas divisum or choledochal cyst), and systemic infections as the primary cause, based on clinical documentation, laboratory investigations, and imaging findings. Variants of uncertain significance (VUS) were recorded separately but were not considered sufficient alone to define genetically confirmed hereditary pancreatitis.

Genetic testing was performed using the Blueprint Genetics Pancreatitis Panel (version 4, 19 October 2019) Plus Analysis, which includes next-generation sequencing–based sequence analysis and copy number variant (CNV) analysis of the following genes: *APOA5*, *APOC2*, *CFTR*, *CPA1*, *CTRC*, *GPIHBP1*, *PRSS1*, *SPINK1*, and *UBR1*. The panel targets protein-coding exons, exon–intron boundaries (±20 bp), and selected non-coding deep intronic variants. Variant classification as pathogenic, likely pathogenic, or VUS was performed according to ACMG/AMP criteria, as reported by the testing laboratory. These genes were selected because of their established involvement in pediatric ARP, CP, and hereditary pancreatitis through mechanisms such as intrapancreatic trypsin activation (*PRSS1*, *SPINK1*, *CTRC*) and ductal or secretory dysfunction (*CFTR*), while *CPA1* and other panel genes have been associated with pancreatitis susceptibility in pediatric cohorts [1,2,6].

Genomic DNA was extracted from peripheral blood samples collected in EDTA tubes. DNA extraction and sequencing were performed by the certified genetic laboratory according to standardized protocols validated for clinical diagnostic use.

Inheritance information was extracted from the genetic laboratory reports, where the reported mode of inheritance reflects established gene-level transmission patterns described in the literature. This designation does not necessarily indicate confirmed familial segregation in the individual patient. Family history of pancreatitis or related pancreatic disease in first-degree relatives was documented when available; however, systematic parental genetic testing was not performed.

Pancreatitis severity was classified as mild, moderately severe, or severe according to the NASPGHAN Pancreas Committee criteria [7]. Briefly, mild AP was defined by the absence of organ failure and local or systemic complications; moderately severe AP included transient organ failure (<48 h) and/or local or systemic complications without persistent organ failure; severe AP was defined by persistent organ failure (>48 h), with or without local complications such as pancreatic necrosis.

Patients were followed clinically from the time of first documented pancreatitis episode until the last recorded visit within the study period. Follow-up duration was calculated from first presentation to the most recent clinical evaluation available in the medical record.

Given the extremely small cohort size (*n* = 11), all statistical analyses were conducted strictly for descriptive and hypothesis-generating purposes. No confirmatory inference was intended. Categorical variables were summarized descriptively, and where statistical tests were applied, results should be interpreted with caution due to sparse data and limited statistical power. Continuous variables were summarized using measures of central tendency and range.

Exploratory computational analyses were performed to examine potential patterns within the dataset. Given the extremely small sample size, these analyses were conducted strictly for illustrative purposes. Detailed methodology and outputs are provided in the Supplementary Material. No predictive modeling, validation, cross-validation, or performance assessment was intended.

Missing clinical variables were handled using complete-case analysis. No imputation procedures were applied due to the small sample size.

During the preparation of this study, the authors used ChatGPT 5.2 (OpenAI) for language editing and grammar correction. The authors reviewed and edited the output and take full responsibility for the content of this publication.

## 3. Results

### 3.1. Demographics and Clinical Characteristics

The mean age of onset of genetically confirmed pancreatitis in the study cohort was 10 years (range: 3–17 years). The sex ratio was M:F = 1.2:1, with males representing 54.55% of patients.

Regarding family history, 45% of patients reported a history of biliary–pancreatic disorders in first-degree relatives. Among these, 60% involved paternal history (chronic pancreatitis and/or pancreatic cancer), while the remaining cases involved maternal biliary pathology (gallstone disease). However, a clearly documented multigenerational pattern of pancreatitis consistent with classical autosomal dominant hereditary pancreatitis was present only in a subset of *PRSS1*-positive patients.

The mode of inheritance recorded in this study reflects gene-level classifications provided in the genetic laboratory reports, which describe established inheritance patterns reported in the literature (e.g., autosomal dominant or autosomal recessive). These classifications do not necessarily indicate confirmed familial segregation in the individual patient. Systematic parental genetic testing or formal segregation analysis was not performed.

Several patients carrying *SPINK1*, *CFTR*, *CTRC*, or *CPA1* variants lacked a positive family history of pancreatitis, consistent with the reduced penetrance and recognized susceptibility or modifier role of these genes rather than uniform vertical transmission.

### 3.2. Genetic Profile

The distribution of identified variants was as follows: *PRSS1* in 38.46% of patients, *CFTR* and *SPINK1* each in 23.08%, *CPA1* in 7.69%, and *CTRC* in 7.69% of patients. Two patients had more than one implicated gene (*SPINK1* + *CFTR* and *SPINK1* + *CTRC*). The average age at first pancreatitis episode was 7.8 years for *PRSS1*-positive patients and 12.6 years for *SPINK1*-positive patients. Detailed variant nomenclature, inheritance pattern, ACMG classification, and variant type are provided in Table 1.

Population allele frequency data were incorporated descriptively in Table 1 to provide additional genetic context. Due to very small cell counts and the inherent limitations of the dataset, the relationship between inheritance pattern and variant classification is presented descriptively without formal inferential interpretation.

Variants are reported using HGVS nomenclature and were classified as pathogenic, likely pathogenic, or variant of uncertain significance (VUS) according to ACMG/AMP criteria, as provided in the accredited diagnostic laboratory reports.

To provide additional genetic context, population allele frequency (AF) data were incorporated from the Genome Aggregation Database, a large-scale reference dataset aggregating exome and genome sequencing data from diverse populations [8]. When allele count (AC) and allele number (AN) values were available in the archived laboratory documentation, AF was calculated directly as AC/AN. In cases where AC/AN values were not retrievable from archived reports, AF values were obtained from curated clinical databases referencing gnomAD [9]. Reported values are approximate where indicated (~) and may vary slightly between database versions and population subsets. Variants not observed in gnomAD are reported as 0 (not observed), whereas entries with AC/AN = 0/0 are reported as not available.

Copy number variant (CNV) analysis was included in the genetic panel; no pathogenic CNVs were identified in this cohort.

In one *PRSS1*-positive case, detailed HGVS-level variant annotation (cDNA and protein nomenclature) was not available in the archived laboratory documentation; however, the presence of a clinically confirmed pathogenic *PRSS1* mutation was clearly documented in the medical record. Given that the primary objective of this study focused on gene-level rather than variant-specific genotype–phenotype correlations, gene-level classification was considered methodologically sufficient for inclusion. Accordingly, this patient was retained in descriptive gene-level analyses.

### 3.3. Disease Severity and Episode Recurrence

**Figure 1:**
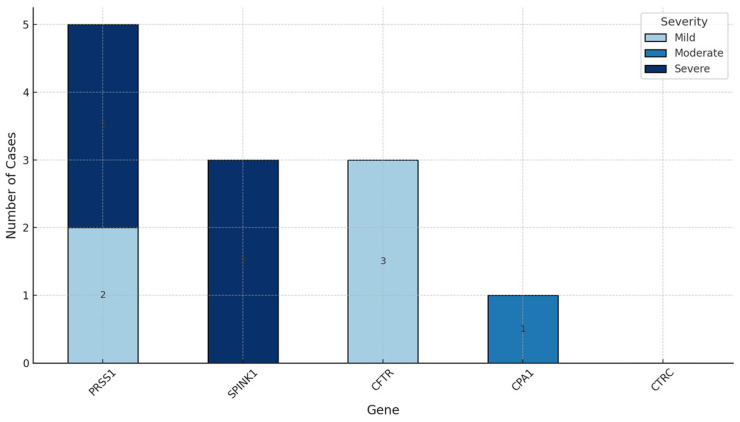
Severity of pancreatitis by involved gene.

Severe pancreatitis was observed in 45.45% of patients, mild pancreatitis in 36.36%, and moderate pancreatitis in 18.18%. Regarding the relationship between severity and the implicated gene, *SPINK1* gene mutation was only associated with cases classified as severe, while for *PRSS1* gene mutation 3 cases were severe, 2 cases were mild and there was no association with moderately severe cases. *CFTR* gene mutation was only associated with mild severity, while *CPA1* gene was linked to a single case of moderate severity, Figure 1.

Distribution of pancreatitis severity (mild, moderate, severe) across different genetic mutations. SPINK1 variants were observed only in severe cases within this cohort. PRSS1 with both mild and severe cases, CFTR with only mild cases, and CPA1 with a single moderate case.

Regarding recurrence, patients with *PRSS1* variants experienced on average 7.4 pancreatitis episodes, whereas those with *SPINK1* variants experienced on average 2.3 episodes. Due to very small group sizes and heterogeneous follow-up, formal between-group inference (e.g., Mann–Whitney U test) was not considered robust and these findings are presented descriptively.

Exploratory computational analyses were conducted to examine potential associations between genetic and clinical variables. Due to the extremely small sample size, these analyses were intended solely to illustrate possible data patterns and should not be interpreted as validated predictors or evidence of predictive performance. Detailed methodology and computational outputs are provided in the Supplementary Material (Supplementary Figure S1).

### 3.4. Surgical Complications

Surgical complications occurred in 54% of patients. Among those with surgical complications, 50% developed pancreatic necrosis and 33.33% progressed to pancreatic pseudocyst. *PRSS1* variants were observed in 66.66% of patients with surgical complications. Given sparse cell counts, the relationship between specific variants and surgical complications is reported descriptively.

Anatomical abnormalities of the Wirsung duct were observed in two patients, both of whom carried the *PRSS1* gene and required duct stenting.

### 3.5. Progression to Chronic Pancreatitis

Patients were evaluated longitudinally between January 2022 and December 2025. Follow-up duration varied depending on the timing of initial presentation within this study interval. Given the retrospective design and limited cohort size, formal time-to-event analysis was not performed.

During the observation period, 63.63% of patients developed imaging-confirmed chronic pancreatitis. Among these individuals, 57.14% carried *PRSS1* variants, 28.57% carried *CFTR* variants, and 14.29% carried *SPINK1* variants. Progression was determined based on documentation of irreversible structural pancreatic changes at the most recent clinical evaluation, rather than at a predefined fixed endpoint.

### 3.6. Exploratory Clustering Analysis

Exploratory clustering analyses suggested the presence of two descriptive patient groupings based on age at onset, recurrence burden, and genetic background. Given the extremely limited cohort size, these groupings should be interpreted strictly as illustrative patterns rather than validated subgroups. Detailed clustering methodology and visualization are provided in the Supplementary Material (Supplementary Figure S2).

## 4. Discussion

Hereditary pancreatitis (HP) is primarily attributed to mutations in the *PRSS1* gene, which encodes cationic trypsinogen and is inherited in an AD pattern with incomplete penetrance. Other implicated genes include *SPINK1*, *CFTR*, *CTRC*, and *CPA1*, which contribute variably to disease pathogenesis [10,11]. These mutations typically lead to premature activation of pancreatic enzymes, resulting in pancreatic inflammation and tissue damage [12]. It is important to distinguish between classical autosomal dominant hereditary pancreatitis, most strongly associated with *PRSS1* mutations, and genetically complex pancreatitis involving susceptibility or modifier genes such as *SPINK1* and *CFTR*. While these variants may significantly influence disease severity, recurrence, or age at onset, they do not uniformly confer a fully penetrant hereditary phenotype [13]. Therefore, the present cohort includes both classical hereditary pancreatitis cases and genetically predisposed pancreatitis cases with modifier gene involvement.

Our study identified pathogenic mutations in several key genes associated with hereditary pancreatitis (HP), with *PRSS1* being the most frequently detected mutation, followed by *CFTR*, *SPINK1*, *CPA1*, and *CTRC*. Additionally, two patients had compound Het mutations, one carrying *SPINK1* and *CFTR* mutations, and another carrying *SPINK1* and *CTRC* mutations. These findings provide valuable insight into the genetic landscape of HP in our pediatric cohort and highlight key trends observed in other studies.

HP often manifests in childhood with recurrent episodes of acute pancreatitis, with an average age of onset of 7.5 years, although this varies based on the specific mutation and inheritance pattern [11]. The observed age of onset for *PRSS1* mutations in this study, 7.8 years, closely aligns with previously reported ranges [14] but highlights variability between families, as noted by Sossenheimer et al., who reported 58% of affected individuals developing pancreatitis before age 5. For *SPINK1* mutations, the average age of onset has been described as 10.7 years [15], consistent with the 12.6 years observed here.

Our cohort of pediatric hereditary pancreatitis patients indicates a slight male predominance. This finding contrasts with some previous studies, which have suggested a higher prevalence of chronic pancreatitis among females [16]. However, our results align with other studies that report no significant sex-based difference in genetic predisposition to hereditary pancreatitis [17]. While the overall sex distribution in hereditary pancreatitis remains debated, these differences may be influenced by sample size, geographic variation, and genetic background.

Our observations are consistent with previously reported associations between certain genetic variants and pancreatitis severity; however, given the limited cohort size, these findings should be interpreted as preliminary and hypothesis-generating. Specifically, *PRSS1* mutations were linked to severe outcomes, with three severe and two mild cases identified, aligning with their reported association with aggressive disease progression [18]. *SPINK1* mutations were linked exclusively to severe cases in our research, further supporting their role as significant risk factors [10,19]. However, this observation should be interpreted with caution, as *SPINK1* is widely regarded as a disease-modifying or risk-enhancing gene rather than a primary causative driver. Genotype–phenotype relationships in *SPINK1*-associated pancreatitis are likely influenced by coexisting genetic variants, environmental exposures, and epigenetic mechanisms, which may modulate disease severity and clinical expression [1,10,20]. *CFTR* mutations, often described as modifiers, were linked exclusively to mild cases, consistent with their less severe clinical impact. *CPA1* mutations, which are rarely associated with pancreatitis [21], were linked to a single moderate case in this cohort. Two patients in our cohort carried mutations in two different genes (*SPINK1* + *CFTR* and *SPINK1* + *CTRC*), which is a significant finding. These cases support the concept of gene–gene interactions, whereby the coexistence of multiple pancreatitis-associated variants may synergistically amplify disease susceptibility, contribute to earlier onset, and increase the risk of a more severe or progressive clinical course.

The analysis of family history suggests a hereditary component, particularly among *PRSS1*-positive patients, in whom a paternal history of chronic pancreatitis or pancreatic cancer was more frequently reported, consistent with the established association between *PRSS1* variants and classical autosomal dominant hereditary pancreatitis [12,18]. However, this observation is based on documented clinical history rather than systematic parental genetic testing or formal segregation analysis.

Maternal history more commonly involved biliary conditions such as gallstone disease, suggesting hepatobiliary clustering rather than confirmed genetic transmission [10]. In contrast, several patients carrying *SPINK1* and *CFTR* variants lacked a clearly documented family history of pancreatitis, consistent with the reduced penetrance and modifier role of these genes, which are frequently encountered in genetically complex or sporadic cases [6,10]. The limited and heterogeneous family history patterns observed in this cohort further highlight the distinction between classical *PRSS1*-associated autosomal dominant hereditary pancreatitis and susceptibility-associated pancreatitis involving modifier genes.

Exploratory clustering analysis using K-Means and PCA suggested potential internal pattern differentiation within the cohort. However, given the extremely small sample size and the absence of formal cluster validation, these observations should be interpreted strictly as descriptive and hypothesis-generating rather than as evidence of clinically meaningful patient subtypes or validated stratification models [1]. Surgical complications were significant, with 54% of patients affected. *PRSS1* mutations were implicated in most severe cases, including pancreatic necrosis (50%) and pseudocysts (33.33%) consistent with reports linking *PRSS1* mutations to severe chronic pancreatitis complications, such as necrosis, pseudocysts, and ductal calcifications, often requiring surgical interventions [22]. Additionally, two *PRSS1*-positive patients exhibited Wirsung duct anomalies, necessitating duct stenting, a finding similarly reported in the literature [23].

Interestingly, one patient with a *SPINK1* mutation experienced pancreatic necrosis, diverging from its typical characterization as a modifier associated with milder disease. This finding underscores that, although *SPINK1* variants are generally associated with milder phenotypes, severe complications may occasionally occur, likely reflecting complex interactions between genetic susceptibility and environmental or inflammatory modifiers [1,23]. Panchoo A et al.’s review reinforces the role of *SPINK1* mutations as risk enhancers rather than independent causative agents, with occasional severe outcomes [6,23].

In our cohort, 63.63% of patients developed chronic pancreatitis during the study period. This proportion should be interpreted with caution, as the study was conducted in a tertiary referral center and follow-up duration was heterogeneous.

A study from China, conducted on a cohort of 276 children, emphasizes the significant role of genetic mutations in the progression from acute pancreatitis (AP) to acute recurrent pancreatitis (ARP) and CP in children. Mutations in genes such as *PRSS1*, *SPINK1*, *CFTR*, and *CTRC* were identified as contributing factors. Notably, *PRSS1* mutations were more closely associated with CP and disease progression than *SPINK1* mutations [24].

In a study by Kumar et al., mutations in *PRSS1* and *SPINK1* genes were found to influence the development of CP. The study also suggests that ethnicity may play a role in disease progression [17].

David S. Vitale et al. discusses the prevalence of genetic mutations in pediatric CP cases, reporting that genetic testing was positive in 68–70% of children with CP, with mutations commonly found in genes like *PRSS1*, *SPINK1*, *CFTR*, and *CTRC* [25].

This study has several limitations that should be acknowledged. First, the relatively small cohort size and retrospective design limit the generalizability of the findings and preclude robust statistical inference. The machine learning analyses were exploratory and hypothesis-generating in nature and should not be interpreted as validated predictive models. Second, genetic interpretation relied on archived diagnostic reports, and although population allele frequency data were incorporated for additional context, complete AC/AN data were not uniformly available for all variants. Finally, although viral infections such as pediatric COVID-19 and hepatitis B or C have been associated with systemic inflammatory alterations [26,27], infectious exposures were not systematically evaluated in our cohort; therefore, no conclusions regarding their influence on hereditary pancreatitis severity or recurrence can be drawn. Prospective studies incorporating standardized infection history, inflammatory biomarkers, and longitudinal follow-up are needed to further clarify these interactions.

The results of this study highlight the necessity of considering inheritance patterns when classifying genetic variants in HP. Understanding these associations may lead to improved genetic counseling and better risk stratification for affected families. Further studies with larger cohorts are needed to validate these findings and explore potential mechanisms underlying the observed relationship between inheritance and pathogenicity. Although computational exploratory approaches may offer insights into complex genotype–phenotype relationships, their application in very small cohorts requires extreme caution and independent validation before any clinical interpretation or translation can be considered.

Genetic testing plays an important role in early diagnosis, personalized management, and prevention of hereditary diseases like pancreatitis, enabling better outcomes by identifying at-risk individuals, guiding treatment, and mitigating complications. Although access to genetic testing in Romania has improved in recent years, availability remains heterogeneous and may be limited by resource constraints and the uneven distribution of specialized genetic services, with some analyses still requiring external laboratory support. These factors should be considered when translating genotype-informed strategies into routine clinical practice, particularly in resource-limited settings. Genetic testing in our hospital has made notable progress over the past years, particularly in the diagnosis and management of hereditary pancreatitis and other rare diseases, and it is steadily advancing, improving access and integration despite challenges in cost and awareness.

## 5. Conclusions

In this small Romanian pediatric cohort with genetically confirmed pancreatitis associated with pathogenic variants in pancreatitis-related genes, *PRSS1* variants were the most frequently identified, followed by *CFTR*, *SPINK1*, *CPA1*, and *CTRC*. The observed patterns suggest that *PRSS1-* and *SPINK1*-positive patients may present at an earlier age and experience a higher disease burden, whereas *CFTR* and *CPA1* variants may be associated with milder clinical phenotypes; however, these trends require validation in larger, independent cohorts.

Family history remained clinically informative for etiological classification and counseling, although inheritance designations reflected gene-level laboratory reporting rather than confirmed segregation.

The identification of compound or multi-gene variants highlights the potential role of gene–gene interactions in pediatric pancreatitis and supports further investigation of their interplay with environmental and inflammatory modifiers.

While the exploratory analyses provide descriptive insight into genotype–phenotype variability, further multicenter studies with adequately powered longitudinal designs are required before clinical stratification frameworks can be proposed.

## Figures and Tables

**Table 1:** Genetic mutation distribution and clinical characteristics of the study cohort.

Gene	cDNA Change	Protein Change	Reported Gene-Level Inheritance	ACMG Classification	Phenotype	Variant Type	Zygosity	AF
***CFTR***	c.1624G > T; c.613C > A	p.(Gly542Ter); p.(Pro205Thr)	AR	P	HP, CF	Nonsense; Missense	Two het variants	~0.0003 (0.03%); Not available
***CFTR***	c.1521_1523del	p.(Phe508del)	AR	P	CFTR-RD	In-frame deletion	Het	~0.0074 (0.74%)
***CPA1***	c.1145G > A	p.(Arg382Gln)	AD	VUS	HP	Missense	Het	0.00003 (0.003%)
***PRSS1***	c.365G > A	p.(Arg122His)	AD	P	HP	Missense	Het	0.000011 (0.0011%)
***PRSS1***	Variant not available	Variant not available	AD	P	HP	Not available	Het	Not applicable
***PRSS1***	p.A16V	p.(Ala16Val)	AD	P	HP	Missense	Het	~0.00009 (0.009%)
***PRSS1***	c.311T > C	p.(Leu104Pro)	AD	P	HP	Missense	Het	0 (not observed in gnomAD)
***PRSS1***	c.311T > C	p.(Leu104Pro)	AD	P	HP/TP	Missense	Het	0 (not observed in gnomAD)
***SPINK1***	c.194 + 2T > C	-	AD	P	HP	Splice-site	Het	0.00030 (0.030%)
***SPINK1* + *CFTR***	c.194 + 2T > C/c.4379C > T,	p.(Ser146Phe)	AD, AR	P/VUS	HP/CF, CBA	Splice-site	Het	0.00030 (0.030%); Not available
***SPINK1* + *CTRC***	c.194 + 2T > C/c.738_761del	p.(Lys247_Arg254del)	AD	P	HP	Splice-site	Het	0.00030 (0.030%); 0.00008 (0.0078%)

Abbreviations: AD—autosomal dominant; AR—autosomal recessive; AF—allele frequency; ACMG—American College of Medical Genetics and Genomics; CF—cystic fibrosis; CFTR-RD—CFTR-related disorder; CBA—congenital bilateral absence of the vas deferens; HP—hereditary pancreatitis; Het—heterozygous; VUS—variant of uncertain significance; gnomAD—Genome Aggregation Database.

## Data Availability

The data presented in this study are available on request from the corresponding author.

## Supplementary Materials

The following supporting information can be downloaded at: https://www.mdpi.com/article/10.3390/jcm15051779/s1, Figure S1: Exploratory Computational Analysis Outputs; Figure S2: Exploratory Clustering Visualization.

## Abbreviations

The following abbreviations are used in this manuscript:

AD	autosomal dominant
ACMG	American College of Medical Genetics and Genomics
AMP	Association for Molecular Pathology
AP	acute pancreatitis
ARP	acute recurrent pancreatitis
CBA	congenital bilateral absence of the vas deferens
CF	cystic fibrosis
CFTR	cystic fibrosis transmembrane conductance regulator
CNV	copy number variant
CP	chronic pancreatitis
CPA1	carboxypeptidase A1
CTRC	chymotrypsin C
CFTR-RD	CFTR-related disorder
HGVS	Human Genome Variation Society
HP	hereditary pancreatitis
INSPPIRE	International Study Group of Pediatric Pancreatitis: In Search for a Cure
LASSO	least absolute shrinkage and selection operator
NASPGHAN	North American Society for Pediatric Gastroenterology, Hepatology and Nutrition
PCA	principal component analysis
PRSS1	protease, serine 1 (cationic trypsinogen)
SPINK1	serine protease inhibitor Kazal type 1
TP	tropical pancreatitis
VUS	variant of uncertain significance
