# Supplementary material for: Genotype-Phenotype Correlations in Pediatric Hereditary Pancreatitis: Evidence from a Romanian Retrospective Cohort
Source: J Clin Med. 2026 Feb 26;15(5):1779. doi: 10.3390/jcm15051779 (PMC12985834; doi:10.3390/jcm15051779)
Supplement: Supplementary file 1 [file jcm-15-01779-s001.zip › jcm-4108310-supplementary.pdf]

Supplementary Methods

Exploratory computational analyses were performed to explore potential genotype–phenotype patterns within the dataset. Decision tree modeling, LASSO logistic regression, principal component analysis (PCA), and K-means clustering were applied without model validation, cross-validation, or performance assessment. These analyses were conducted exclusively for illustrative purposes and were not intended for predictive modeling or clinical inference.

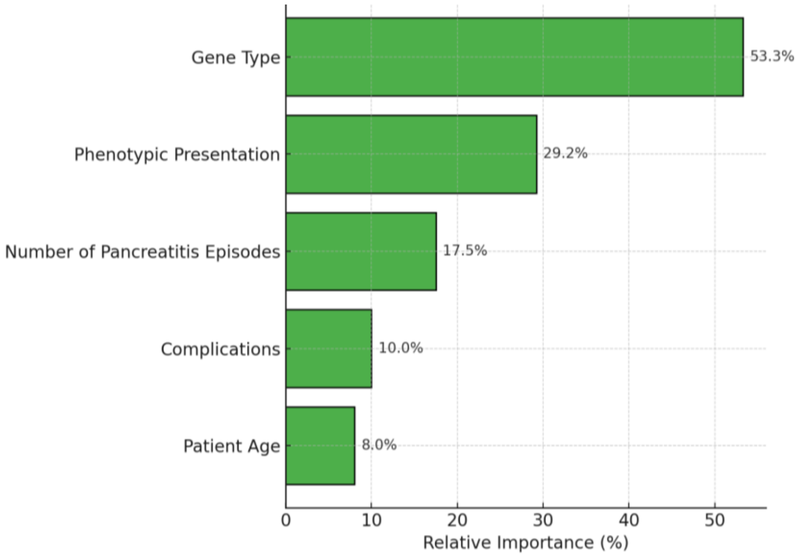

Supplementary Figure S1. Exploratory Computational Analysis Outputs.

This figure illustrates the results of exploratory decision tree and LASSO regression analyses. Given the extremely small sample size (n = 11), these outputs are provided for descriptive visualization only and should not be interpreted as stable predictive measures.

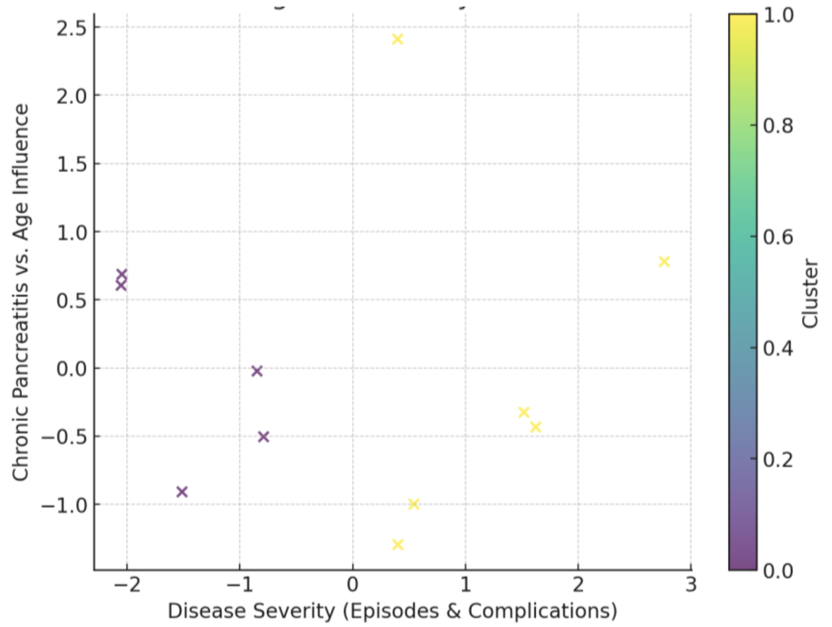

Supplementary Figure S2. Exploratory Clustering Visualization.

This figure presents the results of PCA and K-means clustering performed for illustrative pattern recognition. No formal cluster validation or performance metrics were applied. Identified groupings should be interpreted strictly as descriptive visual patterns.
